# Lachrymal Obstruction: Its Results, Dangers and Treatment
1Read at the meeting of the Bristol Medico-Chirurgical Society, March 11th, 1908.


**Published:** 1908-06

**Authors:** Charles Goulden

**Affiliations:** Demonstrator of Anatomy in University College, Bristol


### LACHRYMAL OBSTRUCTION: ITS RESULTS, DANGERS AND TREATMENT.[1]

BY

CHARLES GOULDEN, M.A., M.B., M.C. (Cantab.), F.R.C.S. (Eng.),

*Demonstrator of Anatomy in University College, Bristol.*

---

THE lachrymal apparatus consists essentially of the lachrymal gland which secretes the tears, and the canaliculi which collect them from the surface of the globe, and the lachrymal sac and duct which conduct them to the nasal cavity.

With the lachrymal gland we are not immediately concerned, and hence the considerations which follow will be confined to the lachrymal sac and nasal duct.

The canaliculi commence as two minute transversely oval apertures placed just behind the summit of the lachrymal papillæ, which are situated at the inner end of both upper and lower lid. These puncta open into the canaliculi, which are united with one another, internal to the caruncle, and open into the outer and fore part of the lachrymal sac. The canaliculi are lined by stratified epithelium on a basement layer, and have on their walls striped muscle fibres derived from the tensor tarsi muscle arranged spirally, but near the papilla circularly so as to form a sphincter; the lachrymal sac is situated in the lachrymal groove, and forms the expanded upper extremity of the lachrymal passage, measuring 12—15 mm. in length, 7 mm. antero-posteriorly, and 4 mm. transversely. It lies in a splitting of the periosteum, which divides along the crest of the lachrymal bone. The posterior layer covers the floor of the lachrymal groove, and the anterior becomes the internal palpebral ligament.

The upper blunt and blind end of the sac rises about 2 mm.

[1] Read at the meeting of the Bristol Medico-Chirurgical Society, March 11th, 1908.

above the upper edge of the internal palpebral ligament; externally it receives the canaliculi guarded by the valve of Rosenmuller, and below it opens into the nasal duct, its down ward continuation. Over it lie the palpebral ligament and a part of the orbicularis muscle.

Where the sac narrows to become the nasal duct there is an annular fold based upon a thickened ring of periosteum ; this fold is the valve of Krause. The sac is lined with ciliated epithelium and mucous secreting cells ; the submucosa is loosely tied down to the periosteum, and is surrounded by a cavernous system of veins continuous with the vessels of the inferior nasal meatus.

The nasal duct is about 18 mm. in length and 3—4 mm. in diameter. It lies in a groove in the superior maxilla, and running downwards and backwards opens into the inferior meatus of the nose at the junction of the anterior with its posterior three-fourths, about 30 mm. behind the posterior margin of the nostril. Its lower extremity is guarded by the valve of Hasner.

The mucous membrane of the sac and duct forms one continuous whole, and there is, therefore, no dividing line between these two structures. The main distinction is the fact that the sac is surrounded on one side only by bone, and hence can enlarge in other directions, whereas the duct is entirely surrounded by a bony casing. Hence, if the lachrymal channels be distended with fluid, the only place where this can show itself externally is by a visible enlargement of the sac just below the internal palpebral ligament.

The nasal duct is the place where strictures occur, which never occur in the sac, and only occasionally in the canaliculi.

The lachrymal secretion is a clear alkaline fluid containing about 1 per cent. of solids, chiefly sodium chloride. Normally, the amount of tears secreted is not more than is lost from the surface of the globe by evaporation, only a minute quantity escaping into the lachrymal passages. When excessive, as by the irritation of a foreign body, the surplus passes into the nose, and becomes apparent by the constant sniffing and blowing of the nose.

The eye is not entirely dependent upon the lachrymal gland for its moisture ; a certain amount is derived from the secretion of the conjunctiva and the several accessory glands present, so that even when the lachrymal gland is removed or degenerates the eye does not become dry.

In the conduction of the tears the features to be considered are the series of events that occur with each act of winking, and the inherent elasticity of the lachrymal sac.

With each act of winking the lid margins come into contact in a continuous wave commencing from their outer part, that is, the outer part of the lids meet first, and this meeting travels from without to the inner parts of the lids. The palpebral fissure also moves bodily inwards. Thus any superfluous tears are swept towards the triangular area situated over the caruncle. That the proper closure of the lids is essential is shown by the epiphora, which is such a constant feature in paralysis of the facial nerve.

With each contraction of the orbicularis muscle tension of the internal palpebral ligament is produced, and also a contraction of Horner's muscle, which lies behind the lachrymal sac. This causes the anterior and posterior walls of the sac to be separated, and thus expands its cavity. Again, the puncta are drawn into closer contact with the globe, and no doubt capillarity comes into play ; in this way tears are sucked into the lachrymal sac. The return of tears is prevented by the circular and spiral musculature of the canaliculi and the imperfect valvular apparatus of the mucous membrane of the nasal duct.

When the lids are opened again the walls of the sac come together, and thus by elastic recoil, assisted by the action of gravity on the tears, force the fluid down the lachrymal duct. This conclusion is supported by the fact that in dilatation of the sac, and its subsequent atony from distension, the sac is not emptied even when the nasal duct is patent.

Again, since lachrymal secretion finds its way into the lachrymal sac when the nasal duct is occluded, the hypothesis that descent of tears is due to the suction caused by the act of inspiration cannot be accepted.

Obstruction to the nasal duct—which forms the starting-point of all the troubles met with in considering the lachrymal passages—is often secondary to affections of the nasal cavity.

They are :—

(1) Inflammation of the mucous membrane of the nose. If swelling of the nasal mucous membrane exists, it is easy for the opening of the nasal duct into the inferior meatus to be occluded, or for the swelling to extend far up the nasal duct, and such a condition would follow an ordinary cold in the head. It may also occur in chronic inflammatory diseases of the nose, such as chronic rhinitis, but in such a condition as ozæna the duct is occluded by cicatricial contraction.

(2) Ulceration of the nose due to tuberculosis and syphilis.

One of the commonest causes of nasal obstruction is congenital syphilis, when this disease attacks the nose; and besides the ulceration of the mucous membrane, it is usual to meet with caries of the bones encasing the lachrymal sac and duct.

(3) Tumours of the nose, the commonest being polypi. The simplest form of lachrymal obstruction is the obliteration of the nasal duct due to swelling of the mucous membrane, and the surrounding plexus of veins, due to the extension of a similar condition from the nose. Here there is no organic stricture, and as the coryza subsides so does the lachrymal obstruction disappear. However, when chronic the condition becomes a great annoyance, owing to the epiphora and the frequency with which the patient has to dry his eye. This is the simplest condition for which patients usually seek treatment.

The next step is that of chronic dacryocystitis, or lachrymal mucocele. The patient complains of epiphora; we find a soft swelling just below the internal tarsal ligament, due to over-filling of the lachrymal sac, and upon pressure the contents are evacuated through the puncta into the conjunctival sac. This consists either of a puriform fluid, or in cases of very long standing clear mucus. The contents may in some cases be evacuated into the nose, but this is exceptional.

Associated with this condition, it is usual to find a stricture of the nasal duct. This occurs most commonly at the place where the lachrymal sac joins the nasal duct, where, as was previously stated, the wall of the duct is constricted by a ridge of the surrounding periosteum. As a result of this stricture, the secretions of the conjunctiva are retained in the sac, and the lachrymal sac becomes gradually filled, and here the contents remain stagnant and undergo decomposition. The conjunctival sac swarms with microbes, and these are swept into the lachrymal sac, and here flourish on the dead material present. The lining membrane of the sac becomes irritated and inflamed, its mucous secretion is greatly increased—as is seen in chronic conjunctivitis—and the sac wall ultimately exudes a purulent secretion much resembling pure pus.

The tumour of the distended sac may grow to quite large dimensions, and one as large as a Barcelona nut is not very uncommon.

The course of the condition is always extremely chronic, and in the majority of cases incurable. If left to itself, the sac may undergo atrophy, and thus a cure be effected.

Lachrymal mucocele is sometimes seen in babies, and this is due to the blocking of the lower end of the nasal duct by epithelial debris, or incomplete canalisation of the rod of cells from which the nasal duct is developed.

*Acute Dacryocystitis, Lachrymal Abscess.*—It is but a small step from the condition of mucocele to the next seen in acute dacryocystitis. If some of the contents of the sac escape into the surrounding tissues, either through some ulceration of the mucous membrane or an abrasion caused by probing, a cellulitis of the surrounding tissues follows, and the patient will present himself with great swelling of the lids and face, and even chemosis of the conjunctiva ; fluctuation will be obtained later over the area of the sac, and if left to itself the abscess will burst just below the internal tarsal ligament. As the result a sinus is formed, connecting the interior of the sac with the skin of the face, and the secretions will escape by this path. If the sinus close, a recurrence of the inflammation follows, and a succession of lachrymal abscesses.

*The Dangers of Lachrymal Obstruction.*—It may appear that apart from the annoyance caused by the epiphora, the disease may be considered quite a trifling one, but this is far from the truth, and, especially in hospital practice, we are constantly seeing eyes hopelessly damaged as a result.

One of the most serious of all diseases of the eye is serpiginous ulcer, or hypopyon keratitis, due to an invasion of the cornea by the pneumococcus. The sequence of events is this :—

The patient has lachrymal obstruction, and the resulting mucocele is constantly discharging its contents into the conjunctival sac. As previously stated, this discharge is full of organisms, including the pneumococcus ; some slight injury to the cornea occurs, an abrasion may be due to a touch or the presence of a foreign body, and the wound becomes infected. If this occurs, the best that can be hoped for is a corneal scar, which more or less seriously reduces vision.

A common result is perforation of the cornea by ulceration, and a dense leucoma with inclusion of the iris, causing the eye to become for all practical purposes blind, and unless judiciously treated excision will be required for the pain produced by a secondary glaucoma. The other alternative is panophthalmitis, and even sympathetic ophthalmia is not unknown.

It is, therefore, the invariable practice to examine for the presence of a mucocele in any patient with corneal ulceration, since it is obviously of no avail to treat an ulcer in the presence of a focus of certain reinfection. Again, the lachrymal sac must always be examined in any patient who requires operation, be it for squint or any intra-ocular operation, such as extraction of cataract or glaucoma iridectomy. To undertake an operation in the presence of a mucocele is to court disaster.

The diagnosis of lachrymal mucocele is easy when the sac is full of pus which can be easily and visually expressed, but when any doubt exists two methods are available.

If a drop of fluorescin be dropped into the conjunctival sac, and the patient be directed to open and close his eye several times ; the presence of fluorescin on his handkerchief when he blows his nose will be proof of the patency of the nasal duct. It does not necessarily follow that the failure of this test proves the patient to have lachrymal obstruction.

The other method is, after dilating the lower canaliculus, to inject some fluid into the sac by means of a special syringe. If this be done with the patient's head bent forward, the fluid will escape through his nose if the duct be patent ; if the head be bent backwards, ask the patient if he feels any fluid trickling down the back of his throat. This is often apparent by the swallowing movements made.

*The Treatment of Lachrymal Obstruction.*—In the treatment of lachrymal obstruction it will be necessary to consider the various stages through which the condition passes, and in what stage the patient presents himself for treatment.

There is a condition causing epiphora, and although not due to blocking of the nasal duct, yet is functionally comparable; it is want of proper approximation of the lower punctum to the globe. It is a common practice in such a condition to slit the lower canaliculus, and if it be deemed insufficient, to remove part of its posterior wall with scissors ; this may remove the epiphora, but a better method is to bring the punctum back to its original position, and avoid unnecessary mutilation. If the point of the galvano-cautery be applied about 3 mm. behind and below the punctum, and the lid seared fairly deeply, the resulting cicatricial contraction will draw the punctum back into its place. It is quite a painless operation under cocaine, and may easily be repeated.

In cases of true obstruction, any abnormal nasal condition should receive treatment, and this is a point likely to be overlooked.

In those cases in which the obstruction is due to swelling of the mucous membrane, and in a chronic condition, the sac should be syringed daily with boracic acid lotion, followed by 15 per cent. protargol or sulphate of zinc lotion, and after a few moments again washed out. These cases improve greatly, and may be cured by this simple method. If, however, no improvement follow after, at the most, six weeks, the condition is probably one of organic stricture of the duct.

So far only cases of simple obstruction without mucocele have been considered. If in a patient with or without mucocele a course of syringing as above described produces no marked improvement in six weeks—and it is a treatment patients may be quite easily taught to carry out for themselves—the lachrymal sac should be excised.

In babies with mucocele a mode of treatment based on the physiological action of the various factors that empty the sac is most successful. The child is placed on its back, and a pool of boracic lotion is allowed to rest over the inner canthus; alternately the outer canthus is pulled upon and the lachrymal sac compressed, thus exaggerating the normal conditions seen when the eye is opened and closed voluntarily. The sac should not be syringed, since even with the greatest care extravasation of fluid frequently results, and even facial cellulitis. This should be repeated three or four times daily, and can easily be performed by the mother or nurse.

In acute dacryocystitis the abscess should be incised, and when all inflammatory signs have disappeared the sac should be excised.

It will be noticed that no mention whatever has been made of the usual treatment of lachrymal obstruction by the use of probes, the insertion of solid or hollow styles, and the rest. The objections to this method are many, and some may be mentioned here:—(1) Before the uses of probes the lower canaliculus has to be slit; (2) in cases of obstruction due to congestion of the mucous membrane the practice is not only unnecessary but also unsurgical; (3) it is extremely painful, and the chances of damage to the mucous membrane are considerable, especially in cases due to congestion, when the obstruction may be aggravated; (4) it is uncertain, and in cases in which the treatment has been carried out for months, or even years, a failure is the frequent result; (5) in cases in which a stricture exists, the passage of probes must injure the mucous membrane, and the stricture may be made worse; (6) the common seat for stricture is completely surrounded by bone, so that a probe cannot stretch it to anything approaching the extent required to overcome the resilience of the fibrous tissue causing the obstruction, and if a probe which would appear sufficiently large to thoroughly dilate the stricture be used, the only result is to crush the walls of the nasal duct between the bone and the probe ; (7) the results of excision of the lachrymal sac are so satisfactory, and the operation so comparatively easy, that the certainty of cure is not to be compared with the great uncertainty of probing.

There is one condition in which it may be allowable to pass a probe, and that but once. In cases of mucocele in quite small children which resist prolonged treatment on the plan above described, the passage of a small probe under chloroform without previously slitting the canaliculus is often followed by a complete cure.

The operation for excision of the lachrymal sac is performed as follows :—

The internal palpebral ligament is put on the stretch, and made prominent by drawing on the external canthus. An incision about 2 cm. in length is then made, commencing just above the centre of the ligament, about 5 mm. from the inner commissure of the lids, and continued downwards and then a little outwards, following the margin of the orbit. Care should be taken not to cut too far inwards, since injury to the angular vein causes troublesome hemorrhage, and obscures the field of operation. The incision should be planned so as to completely divide the internal palpebral ligament and lay bare the sac wall at once, or even open it, and then the wound is made to gape with an automatic retractor. The sac is then grasped in a stout pair of fixation forceps, and the rest of the operation performed with a blunt dissector. The outer wall is first freed, then the fundus, then the inner wall ; the duct should be freed as low down as possible, and either twisted off or divided with a stout sharp pair of scissors. It is then an advantage to scrape the lower part of the nasal duct with a small sharp spoon, although this is not always necessary. Two skin sutures of silkworm gut are then inserted, and a small pressure pad applied. The accompanying oozing of blood is trifling, and the field can be kept dry by a good assistant using dry pledgets of wool, and occasionally temporarily plugging the wound for a few moments with wool soaked in a solution of adrenalin.

The wound heals by first intention, and dressings may be abandoned in about four days.

The succsss of the operation depends upon the complete removal of the sac, and the place where failure may occur is in dealing with the fundus. If, however, the internal palpebral ligament be divided, there should be no difficulty, and before closing the wound it should be rendered anæmic with adrenalin, and well inspected to make sure the sac has been completely removed.

It might be thought that after removal of the lachrymal sac the epiphora would be worse, or at least as bad, as before. Experience shows that patients are grateful for the relief and comfort given them, and that the epiphora disappears and only makes itself apparent in a very sharp wind or in sudden changes of temperature, as when leaving a warm room and going into the night air. The explanation seems to be this. The secretion of the mucocele is a constant source of irritation to the conjunctiva, and in every case a certain amount of conjunctivitis is present, and when the source of irritation is removed, not only does the hypersecretion of tears disappear, but the lachrymal gland seems to accommodate itself to its new conditions.

The scar of the operation falls into the natural folds of the face, and becomes almost invisible, so that in many cases it can only be discovered after a careful inspection.

